# Unlocking the chromatin code by deciphering protein–DNA interactions

**DOI:** 10.15252/msb.20167332

**Published:** 2016-11-11

**Authors:** Dalila Bensaddek, Angus I Lamond

**Affiliations:** ^1^ Laboratory of Quantitative ProteomicsCentre for Gene Regulation and ExpressionSchool of Life SciencesUniversity of DundeeDiscovery CentreDundeeUK

**Keywords:** Methods & Resources, Post-translational Modifications, Proteolysis & Proteomics, Transcription

## Abstract

Characterizing the composition of protein complexes bound to different genomic loci is essential for advancing our mechanistic understanding of transcriptional regulation. In their recent study, Krijgsveld and colleagues (Rafiee *et al*, 2016) report ChIP‐SICAP, a powerful tool for deciphering the chromatin proteome by combining chromatin immunoprecipitation, selective isolation of chromatin‐associated proteins and mass spectrometry.

Chromatin is the dynamic macromolecular complex of DNA, histones and regulatory proteins, and its structure and composition is critical for all DNA‐dependent processes, including transcription, DNA replication and DNA repair. The fundamental feature of chromatin structure involves the wrapping of DNA around nucleosomes, compacted to varying degrees by folding into higher order chromatin structures and the degree of compaction in turn affects the accessibility of DNA loci to regulatory protein complexes and hence modulates gene expression. Chromatin structure is highly dynamic and can be remodelled in response to diverse stimuli through a combination of changes in DNA methylation and histone post‐translational modifications, as well as via the recruitment of different chromatin‐associated protein complexes. Understanding the molecular interactions between chromatin and chromatin‐interacting proteins is key for understanding gene regulation, both in physiological and disease contexts and depends, *inter alia*, upon having sensitive methods for the accurate measurement of the chromatin proteome.

In recent years, a number of mass spectrometry (MS)‐based proteomic methods have been developed to allow unbiased and comprehensive characterization of chromatin and chromatin‐associated proteins. This includes the detection of DNA binding proteins, such as transcription factors, transcriptional regulators and epigenetic modifiers and the measurement of histone post‐translational modification patterns, which can act as determinants of protein binding. Most of these methods rely on the initial enrichment of chromatin components by chromatin immunoprecipitation (ChIP). This pre‐enrichment step can provide the sensitivity required, either to identify changes in the levels of chromatin‐associated proteins present at a specific locus (e.g. a gene promoter), or to determine the binding sites for a specific protein, or protein complex, over the entire genome and to compare how this may vary under different conditions.

However, in common with all immunoprecipitation and related “pull‐down” methods, ChIP‐based assays can suffer from lack of selectivity, resulting in low signal to background noise. In this case, the background noise results predominantly from the contaminating proteins routinely detected because they either have high affinity for the reagents used for immunoprecipitation, or else have affinity for the highly negatively charged DNA backbone, or are simply low affinity binders present at very high abundance. This high background is particularly problematic in combination with the fact that the abundance of many chromatin‐binding proteins is many orders of magnitude lower compared with other proteins. For example, in a recent study, the abundance of the transcription factor c‐Myc was estimated to be ~2,000 copies per cell, while the average abundance of a ribosomal protein was ~3 million copies per cell (Hukelmann *et al*, 2016). The high levels of background proteins that are consistently identified by immunoprecipitation‐based methods have been previously documented, for example in the “protein frequency library” (Boulon *et al*, 2010) and, more recently, in the so called “CRAPome” (Mellacheruvu *et al*, 2013). Another limitation of existing approaches, especially relevant when analysing chromatin‐interacting proteins, is that the interactors of a given protein may differ, depending on whether the protein is either in its chromatin‐bound or soluble state (Li *et al*, 2015).

While as yet there is no accepted gold standard established for chromatin proteomes, several useful methodologies have been developed for affinity enrichment and analysis of chromatin complexes, such as modified ChIP (Lambert *et al*, 2009), chromatin proteomics (ChroP)(Soldi & Bonaldi, 2014), ChIP‐MS (Engelen *et al*, 2015) and rapid immunoprecipitation mass spectrometry of endogenous proteins (RIME) (Mohammed *et al*, 2016). These methods all share the starting step of crosslinking protein complexes to DNA *in cellulo* to facilitate their recovery under harsh extraction conditions, which helps to reduce background levels of co‐isolated contaminants.

In their recent work, Rafiee *et al* (2016) describe a new method for the selective isolation of chromatin‐associated proteins (SICAP), which improves selectivity. The method involves ChIP of DNA‐protein complexes, combined with biotin tagging of DNA, to allow the specific isolation of DNA‐bound proteins on streptavidin beads, followed by MS‐based protein identification (Fig 1). The use of formaldehyde for chromatin crosslinking allows stringent purification conditions to reduce non‐specific background. Importantly, the antibody used to target the bait protein is removed using a combination of reducing agents and ionic detergents (SDS), prior to streptavidin capture of DNA‐bound complexes, in addition to stringent denaturing/high salt washes after streptavidin purification. This was facilitated by processing the samples using the SP3 protocol developed by the same laboratory (Hughes *et al*, 2014), thus avoiding antibody contamination during the subsequent MS analysis step. Consequently, this improves the proteomics readout compared with other protocols in which the DNA‐bound protein complexes are either resolved by SDS–PAGE, or digested on the beads, prior to LC‐MS.

**Figure 1:**
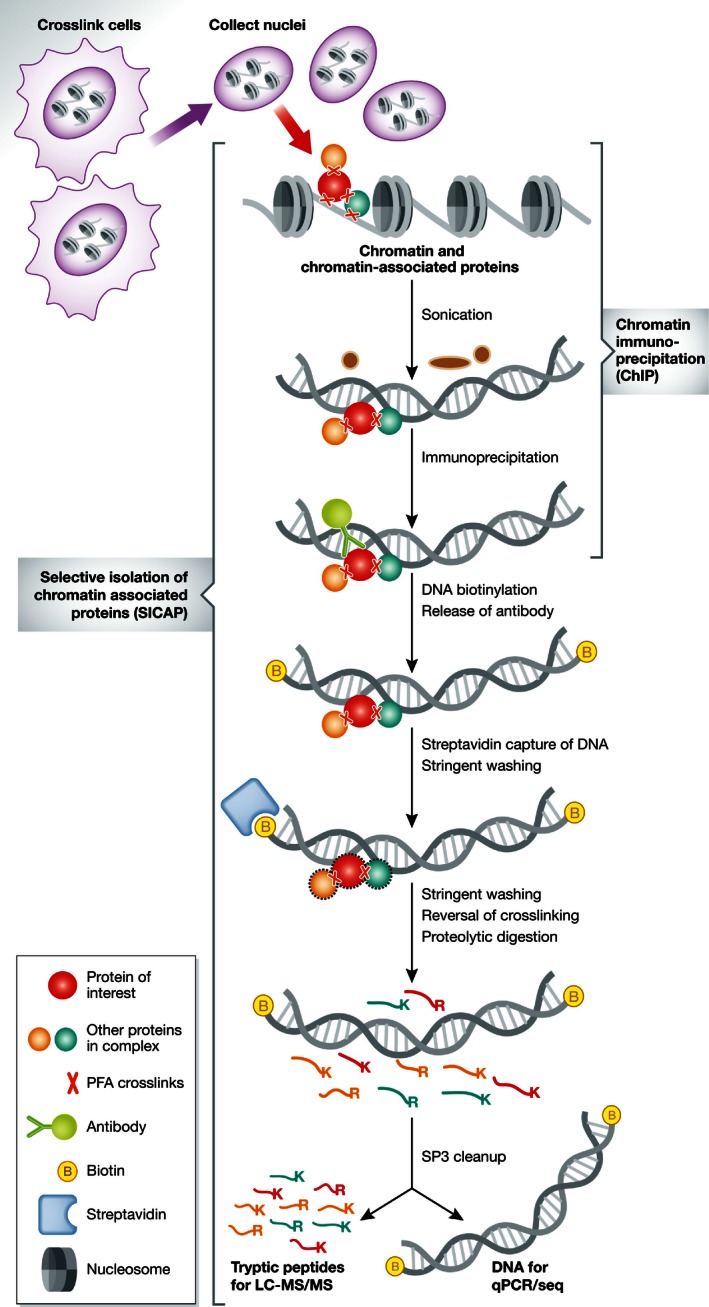
SICAP method Schematic representation of the ChIP‐SICAP workflow, which starts with *in cellulo* formaldehyde crosslinking, followed by sonication and immunoprecipitation of chromatin (ChIP), then by the selective isolation of chromatin‐associated proteins (SICAP). DNA biotinylation allows the removal of the antibody prior to SP3 processing for LC‐MS/MS and ChIP‐seq analyses.

An important feature of ChIP‐SICAP is that it allows the identification of endogenous proteins bound to native chromatin, without the need to over‐express a tagged version of the protein of interest. This prevents, on the one hand, some of the potential artefacts that can arise from over‐expression of tagged protein baits. However, on the other hand, it limits the applicability of the technique to the analysis of proteins for which a high‐quality, “ChIP‐grade”, antibody is available.

The efficacy of the ChIP‐SICAP method was elegantly demonstrated by analysing the protein interaction network of the canonical pluripotency factors, that is Oct4, Sox2 and Nanog, in mouse ES cells. This resulted in the identification of the E3‐ubiquitin ligase, Trim24, as a new component of the pluripotency network. Trim24 was shown to co‐localize with the pluripotency markers at 813 enhancers, including 88 super‐enhancers. Knockdown experiments using shRNA showed that the Trim24 protein is required to suppress developmental genes and to maintain the expression of cell cycle, DNA replication and Polycomb components.

A notable advantage of the ChIP‐SICAP method is that it permits the simultaneous recovery of both proteins *and* DNA in a single experiment. Therefore, in addition to the identification of co‐localizing proteins, the genomic binding sites of the protein of interest also can be identified, from the same sample, by sequencing. Moreover, capturing the interactors of the bait protein in their DNA‐bound state potentially allows distinguishing between interactions that occur either on, or off, chromatin.

While it represents a very useful technical advance for analysing the chromatin proteome, inevitably, the SICAP technique does have some limitations, in particular related to the mass spectrometric readout. As with most MS‐based approaches, the wide dynamic range of protein abundance levels remains a problem that has to be overcome in order to reliably measure low‐abundance species. The authors recognize this limitation and the resulting preferential detection of higher abundance interaction partners in their experiments. In other types of proteomic analyses, a pre‐fractionation strategy is often combined with LC‐MS/MS. This helps to reduce overall sample complexity and thus enhances the detection and quantitation of low‐abundance species that can otherwise be missed when present in more complex extracts with much higher abundant peptides. In principle, therefore, one could envisage applying this strategy also to methods for chromatin proteomics, such as SICAP. For example, using a form of miniaturized, on‐tip pre‐fractionation step with the immunoprecipitated sample, prior to LC–MS/MS.

Overall, this study by Rafiee *et al* (2016) presents a very useful improvement in the methodological toolbox available for the selective purification and identification of protein complexes and protein interaction networks associated with chromatin. The ChIP‐SICAP method offers several specific advantages: (i) it allows selective and reproducible isolation of chromatin‐associated proteins, together with the cognate, bound DNA element, (ii) it offers the possibility to distinguish between protein interactions taking place either, on or off, chromatin, and (iii) it facilitates combining proteomics analysis and ChIP‐seq in a single experiment. We therefore anticipate that the ChIP‐SICAP approach will be widely adopted in future studies for the analysis of diverse gene regulatory networks, both in human cells and model organisms.

## References

[msb167332-bib-0001] Boulon S , Ahmad Y , Trinkle‐Mulcahy L , Verheggen C , Cobley A , Gregor P , Bertrand E , Whitehorn M , Lamond AI (2010) Establishment of a protein frequency library and its application in the reliable identification of specific protein interaction partners. Mol Cell Proteomics 9: 861–879 20023298 10.1074/mcp.M900517-MCP200 PMC2871420

[msb167332-bib-0002] Engelen E , Brandsma JH , Moen MJ , Signorile L , Dekkers DHW , Demmers J , Kockx CEM , Ozgur Z , van Ijcken WFJ , van den Berg DLC , Poot RA (2015) Proteins that bind regulatory regions identified by histone modification chromatin immunoprecipitations and mass spectrometry. Nat Commun 6: 7155 25990348 10.1038/ncomms8155 PMC4455091

[msb167332-bib-0003] Hughes CS , Foehr S , Garfield DA , Furlong EE , Steinmetz LM , Krijgsveld J (2014) Ultrasensitive proteome analysis using paramagnetic bead technology. Mol Syst Biol 10: 757 25358341 10.15252/msb.20145625 PMC4299378

[msb167332-bib-0004] Hukelmann JL , Anderson KE , Sinclair LV , Grzes KM , Murillo AB , Hawkins PT , Stephens LR , Lamond AI , Cantrell DA (2016) The cytotoxic T cell proteome and its shaping by the kinase mTOR. Nat Immunol 17: 104–112 26551880 10.1038/ni.3314 PMC4685757

[msb167332-bib-0005] Lambert J‐P , Mitchell L , Rudner A , Baetz K , Figeys D (2009) A novel proteomics approach for the discovery of chromatin‐associated protein networks. Mol Cell Proteomics 8: 870–882 19106085 10.1074/mcp.M800447-MCP200 PMC2667365

[msb167332-bib-0006] Li X , Wang W , Wang J , Malovannaya A , Xi Y , Li W , Guerra R , Hawke DH , Qin J , Chen J (2015) Proteomic analyses reveal distinct chromatin‐associated and soluble transcription factor complexes. Mol Syst Biol 11: 775 25609649 10.15252/msb.20145504 PMC4332150

[msb167332-bib-0007] Mellacheruvu D , Wright Z , Couzens AL , Lambert J‐P , St‐Denis NA , Li T , Miteva YV , Hauri S , Sardiu ME , Low TY , Halim VA , Bagshaw RD , Hubner NC , al‐Hakim A , Bouchard A , Faubert D , Fermin D , Dunham WH , Goudreault M , Lin Z‐Y *et al* (2013) The CRAPome: a contaminant repository for affinity purification‐mass spectrometry data. Nat Meth 10: 730–736 10.1038/nmeth.2557 PMC3773500 23921808

[msb167332-bib-0008] Mohammed H , Taylor C , Brown GD , Papachristou EK , Carroll JS , D'Santos CS (2016) Rapid immunoprecipitation mass spectrometry of endogenous proteins (RIME) for analysis of chromatin complexes. Nat Protocols 11: 316–326 26797456 10.1038/nprot.2016.020

[msb167332-bib-0009] Rafiee MR , Girardot C , Sigismondo G , Krijgsveld J (2016) Expanding the circuitry of pluripotency by selective isolation of chromatin‐associated proteins. Mol Cell 64: 624–635 27773674 10.1016/j.molcel.2016.09.019 PMC5101186

[msb167332-bib-0010] Soldi M , Bonaldi T (2014) The ChroP approach combines ChIP and mass spectrometry to dissect locus‐specific proteomic landscapes of chromatin. J Vis Exp 86: e51220 10.3791/51220 PMC4166860 24747196

